# ZBP1 contributes to interferonopathies caused by impaired ADAR1 function

**DOI:** 10.1186/s43556-022-00097-w

**Published:** 2022-12-05

**Authors:** Xiuwu Fang, Long Zhang

**Affiliations:** 1 Institutes of Biology and Medical Science, Soochow University, Suzhou, China; 2 International Biomed-X research center, Second affiliated hospital of Zhejiang University School of Medicine, Zhejiang University, Hangzhou, China

Recently, a study published in Nature by Jiao et al [1]. revealed the underlying mechanism of the contribution of Z-DNA-binding protein 1 (ZBP1) to the Z-RNA-dependent activation of type I interferonopathies caused by adenosine deaminase acting on RNA 1 (ADAR1) mutations or deficiency. In contrast to its role in enhancing the phenotype mediated by mutated ADAR1, ZBP1 did not contribute to the interferonopathies induced by TREX1 deficiency. This study aims to show the critical role of ZBP1 in regulating IFN responses triggered by endogenous RNA, which is closely related to its Zα domain (Fig. 1).

**Fig. 1:**
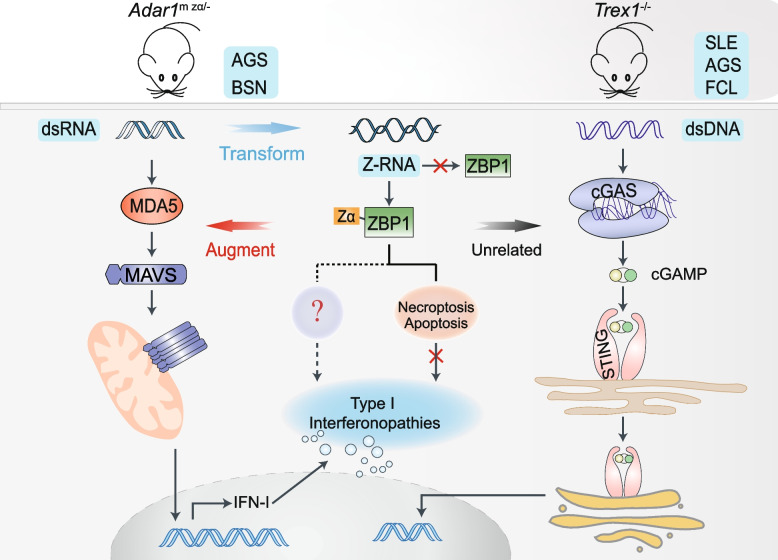
The contributing role of ZBP1 in pathogenic IFN response in the *Adar1*^m zα /–^ model. Hemizygous expression of ADAR1 with a mutated Zα domain resulted in the accumulation of dsRNA and induced a strong MAVS-dependent type I IFN response, causing the severe postnatal lethality phenotype in mice. These dsRNA with the ability to transform into Z-RNA recognized by the Zα domain of ZBP1 augment the type I interferonopathies

Chronic activation of type I interferon (IFN-I) signaling related severe diseases, including Aicardi–Goutières syndrome and bilateral striatal necrosis, is driven by the RNA deaminase ADAR1 mutations; moreover Z-RNA contains an alternative left-handed double-helix structure recognized by the Zα domain of IFN-induced ADAR1, the p150 isoform. Z-RNA and Z-DNA, nucleic acids with a left-handed double-helix structure, are recognized by proteins containing the Zα domain in a conformationally specific manner [2]. Two mammalian proteins, ADAR1 and ZBP1, harbor this particular domain. ADAR1 exists in two forms: P110, which is constitutively expressed in the nucleus, and the cytosolic isoform P150, which contains the Zα domain and is induced by IFN. ADAR1 P150 recognizes and undergoes adenosine-to-inosine base modification of self-RNA fragments produced by endogenous retroelements to prevent the recognition by melanoma differentiation-associated gene 5 (MDA5), leading to pathogenic mitochondrial antiviral signaling (MAVS)-dependent type I IFN responses. Mutations in the Zα domain of ARAR1 cause a deficiency in the P150 isoform, contributing to a range of autoimmune diseases and MDA5-dependent pathogenic IFN responses [3]. ZBP1, an IFN-induced protein, recognizes intracellular viruses and endogenous Z-nucleic acids and induces cell death to trigger antiviral responses, along with tissue damage and inflammation [4]. Previous reports have demonstrated that ZBP1 phosphorylates mixed-lineage kinase-like (MLKL) upon RHIM-dependent-receptor-interacting protein kinase 3 (RIPK3) activation, promoting necroptosis as well as mediating receptor-interacting protein kinase 1 (RIPK1)-engaged Caspase-8-dependent apoptosis. ZBP1 may play a crucial role in the interaction between the ADAR1 Zα domain and Z-nucleic acids, as well as in the prevention of pathogenic IFN responses triggered by ADAR1 mutations [5].

The authors put great effort into generating diverse genetically modified mouse strains. *Adar1*^m zα/m zα^ mice, with two substitutions (N175D/Y179A) in the Zα domain leading to the disruption its interaction with Z-RNA, showed the upregulation of 57 functionally-related IFN-I genes, but did not develop Aicardi–Goutières syndrome pathology. To mimic ADAR1 mutation-associated Aicardi–Goutières syndrome, the group constructed hemizygous *Adar1*^m zα/–^ mice. *Adar1*^m zα/–^ developed the postnatal lethality phenotype accompanied by severe pathology and showed reduced body weight and erythropoiesis ability; they also showed severe small intestine and colon damage compared to that in *Adar1*^m zα/wt^ mice. Many IFN-related genes were upregulated in *Adar1*^m zα/–^ mice. Additionally, *Adar1*^m zα/–^*Mavs*^–/–^ mice did not develop a postnatal lethality phenotype, and grew without displaying pathology. ADAR1 deficiency can induce a pathogenic IFN response in an MDA5-MAVS-dependent manner, which contributes to lethality and pathology. However, genetic-mediated removal of MDA5 rescued the embryonic lethality of mice, these mice still died during the first weeks. This indicated that other immune sensors may contribute to the IFN response caused by ADAR1 deficiency.

The expression of ZBP1 was significantly upregulated in ADAR1 deficient mice. Postnatal lethality was greatly reduced in the *Adar1*^m zα/–^*Zbp1*^–/–^ mice. The elevation of IFN-stimulated genes was strongly inhibited in *Adar1*^m zα/–^*Zbp1*^–/–^ mice compared to that in the control group. However, ZBP1-deficient mice showed weight-loss along with reduced erythropoiesis, intestinal damage and kidney disease. In addition, *Adar1*^m zα/–^*Mavs*^+/–^ mice exhibited a 50% postnatal lethality phenotype, a sharp decrease in body weight and blood values, and increased deteriorative kidney lesions and intestinal damage compared to those in the control littermates. These symptoms were greatly ameliorated in adult *Adar1*^m zα/–^*Mavs*^+/–^ *Zbp1*^–/–^ mice; ZBP1 augments the IFN-I response triggered by ADAR1 deficiency.

TREX1 deficiency leads to the accumulation of self-DNA in vivo and induces activation of the IFN-I response through cyclic GMP-AMP synthase (cGAS)–stimulator of interferon genes (STING) signaling. Unlike the *Adar1*^m zα/–^ model, in which ZBP1 deficiency alleviates the abnormally activated IFN response, ZBP1 failed to regulate the IFN-I levels in the *Trex1*^–/–^ model. These data indicate that ZBP1 specifically recognizes the accumulated RNA driven by ADAR1 deficiency, and mice consequently develop related diseases. ERE-derived transcripts and non-edited RNAs significantly accumulated in ADAR1-deficient mice; and these dsRNAs, which are capable of transforming into Z-RNA, provide ligands for the recognition and activation of ZBP1. ZBP1 Zα domain mutations alleviated the pathological features in ADAR1-deficient mice.

ZBP1 induces RIPK3-dependent FADD-Caspase-8 mediated apoptosis and MLKL-mediated necroptosis. Nevertheless, *Ripk3*^–/–^, *Mlkl*^–/–^, *Fadd*^–/–^*Mlkl*^–/–^, *Fadd*^–/–^*Ripk3*^–/–^ and *Ripk1*^mR/mR^*Mlkl*^–/–^ knockout failed to rescue the pathological phenotype of *Adar1*^m zα/–^mice. Knockout of MAVS and ZBP1 completely prevented the pathogenic IFN response and attenuated intestinal injury in neonatal mice. Based on these results, ZBP1 augmented the pathogenic IFN-I response which was not associated with FADD-Caspase-8-mediated apoptosis, MLKL-mediated necrosis, or RIPK1-mediated proinflammatory response. Although RIPK3 depletion failed to reverse the pathological phenotype of *Adar1*^mZα /–^ mice, RIPK3 knockout resulted in delayed postnatal lethality, consistent with ZBP1 knockout in *Adar1*^–/–^*Mavs* ^–/–^ mice. The underlying mechanism by which ZBP1 promotes the activation of pathogenetic IFN response and development of severe pathology independent of necrosis and apoptosis remains unclear in ADAR1-deficient mice.

This study demonstrates the critical role of ZBP1 in regulating IFN responses triggered by endogenous RNA, which is closely related to its Zα domain. However, some technical issues remain, including assessing the formation of Z-RNA directly in vivo. This study not only discovered a novel mechanism of ZBP1 in regulating IFN signaling independent of necrosis and apoptosis, but also provided a new therapeutic strategy for the clinical treatment of Aicardi–Goutières syndrome and other diseases caused by ADAR1 mutations.

## Data Availability

Not applicable.

## Abbreviations

ADAR1: Adenosine deaminase acting on RNA 1
ZBP1: Z-DNA-binding protein 1
MAVS: Mitochondrial antiviral signaling
MDA5: Melanoma differentiation-associated gene 5
RIPK3: Receptor-interacting protein kinase 3
MLKL: Mixed-lineage kinase-like
cGAS: Cyclic GMP-AMP synthase
STING: Stimulator of interferon genes
cGAMP: Cyclic GMP-AMP
AGS: Aicardi–Goutières syndrome
BSN: Bilateral striatal necrosis
SLE: Systemic lupus erythematosus
FCL: Familial chilblain lupus
